# Current Choroidal Imaging Findings in Central Serous Chorioretinopathy

**DOI:** 10.3390/vision4040044

**Published:** 2020-10-16

**Authors:** Gideon Nkrumah, Dmitrii S. Maltsev, Paez-Escamilla A. Manuel, Mohammed A. Rasheed, Marianno Cozzi, Alessandro Ivernizzi, Marco Lupidi, Sumit Randhir Singh, Jay Chhablani

**Affiliations:** 1 School of Medicine, University of Pittsburgh, Pittsburgh, PA 15213, USA; Gin3@pitt.edu; 2 Department of Ophthalmology, Military Medical Academy, 194044 St. Petersburg, Russia; Glaz.med@yandex.ru; 3 Department of Ophthalmology, University of Pittsburgh Medical Center, Pittsburgh, PA 15213, USA; paezescamillama@upmc.edu; 4 School of Optometry and Vision Science, University of Waterloo, Waterloo, ON N2L 3G1, Canada; ar8mohammed@uwaterloo.ca; 5 Eye Clinic, Department of Biomedical and Clinical Science “Luigi Sacco”, University of Milan, 20122 Milan, Italy; mariano.cozzi88@gmail.com (M.C.); alessandro.invernizzi@gmail.com (A.I.); 6 Department of Biochemical and Surgical Sciences, Section of Ophthalmology, University of Perugia, 06121 Perugia, Italy; marcomed2@gmail.com; 7 Jacobs Retina Center at Shiley Eye Center, University of California, San Diego, La Jolla, CA 92037, USA; sumit.jipmer@gmail.com

**Keywords:** central serous chorioretinopathy (CSCR), CSCR biomarkers, imaging, choroid, optical coherence tomography (OCT)

## Abstract

Background: Central serous chorioretinopathy (CSCR) is a chorioretinal disease affecting mostly middle age males. It is marked by the serous detachment of the neurosensory layer at the macula. This review of the literature provides a framework of the current characteristic/relevant imaging findings of CSCR. Although the pathogenesis of CSCR is unclear, the choroid plays a major role and its changes are fundamental to the diagnosis and treatment of CSCR. Methods: A systematic literature search focusing on current multimodal imaging for CSCR was performed. Only articles reporting on original clinical data were selected, studies in a language other than English were included only if an English abstract was provided. Additional sources included articles cited in the references list of the first selected articles. We deduced imaging findings based on current and relevant literature on the topic. Results: We found that sub foveal choroidal thickness (SFCT) and choroidal vascularity index (CVI) were greater in eyes with acute CSCR than in eyes with chronic CSCR or normal eyes. There was increased choroidal thickness (CT) in the macula compared to peripapillary region. In healthy eyes, the highest CVI was found in the nasal region followed by the inferior, temporal, and superior quadrant. The area with the least CVI was the macula. In eyes with CSCR, 100% had asymmetric dominant vortex veins compared to 38% in normal eyes. Conclusion: Choroidal imaging has advanced the diagnosis of CSCR. This has led to numerous imaging biomarkers like CVI, CT, and hyper-reflective dots for early detection and possible prognostication of CSCR. More techniques like wide field scans and en face imaging are being employed to characterize the choroid in CSCR.

## 1. Introduction

Central serous chorioretinopathy (CSCR) is a disease predominant in middle-age males that affects the choroid and the retinal pigment epithelium (RPE) [1,2]. It is associated with serous RPE detachment with one or more leakage sites resulting in vision loss [3]. A rare bullous form of CSCR has also been described by Sartini, et al. [4]. The exact molecular mechanism is still unclear. CSCR can resolve spontaneously [5], recur or lead to chronic CSCR. The term “central” denotes the effect of vision loss stemming from serous detachments in the macula area. The disease is also associated with pregnancy, steroid use and obstructive sleep apnea [6,7,8].

The pathogenesis of CSCR is still under investigation; however, the mineralocorticoid receptor (MR) pathway has been implicated in the explanation of the disease process [6] as well as genetic predisposition [9]. The mineralocorticoid pathway hypothesis theorizes that under physiologic conditions in ocular tissues, glucocorticoids occupy the mineralocorticoid receptors at basal levels [10] MR activators like aldosterone and corticosterone can cause choroidal vessel dilation and leakage by upregulating an endothelial vasodilatory potassium (K) channel, KCa2.3. Glucocorticoids also act on the ion and water channels in the retinal glial Muller cells [11]. The dysregulation of MR by glucocorticoids contribute to the choroidal vessel leakage and serous retinal fluid accumulation seen on choroidal imaging in CSCR.

The choroid comprises the choriocapillaris, Sattler’s layer and Haller’s layer. Haller’s layer is composed of large choroidal vessels, followed by Sattler’s layer with medium vessels, and smallest innermost choriocapillaris [12]. Measures of different choroidal layers including thickness, volume, and vascularity index (defined as the ratio of the total choroidal vascular luminal area and the total choroidal area on optical coherence tomography scan [13]) have been investigated in the pathogenesis of the disease using different imaging modalities.

**Figure 1:**
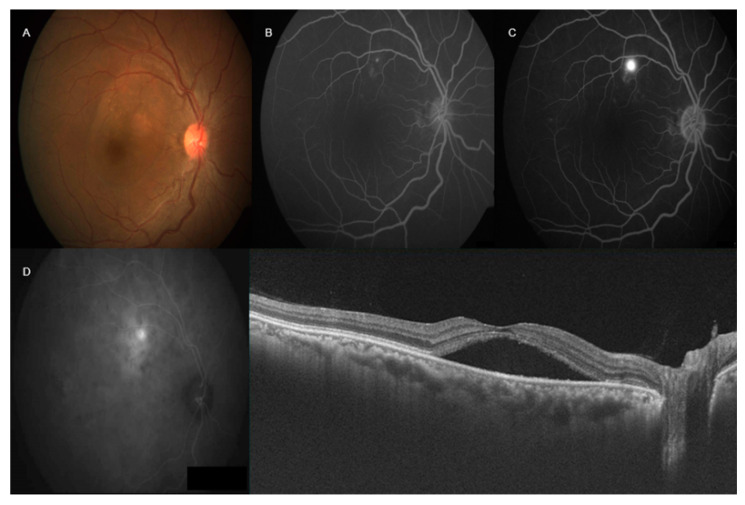
Multimodal imaging in central serous chorioretinopathy (CSCR)—right color fundus photograph (**A**) showed pocket of subretinal fluid involving fovea. Fluorescein angiography (FA) (**B**,**C**) showed ink-blot leakage superior to fovea. Indocyanine green angiography (ICGA) showed increased fluorescence with focal late leakage superior to the fovea (**D**). Swept source optical coherence tomography showed neurosensory detachment involving fovea with increased subfoveal choroidal thickness.

Current evidence in the literature recognizes that CSCR pathogenesis involves choroidal vasculature and its vasomotor control. The accumulation of retinal fluid in CSCR is the result of leakage of fluid through a disrupted retina-choroidal layer facilitated by an underlying choroidal dysfunction. Factors such as choroidal hyperpermeability have been described to explain this process [13]. In addition, leakage sites have been captured by various choroidal imaging techniques like fluorescein angiography (FA) and indocyanine green angiography (ICGA) to facilitate diagnosis (Figure 1) [14]. These imaging methods are described in detail below.

Several choroidal imaging modalities have been introduced to detect and diagnose CSCR [15]. Amongst them are FA, ICGA, adaptive optics-scanning laser ophthalmoscopy (AO-SLO), optical coherence tomography (OCT; swept source (SS) and spectral domain (SD)), and optical coherence tomography angiography (OCTA) [15]. FA imaging can detect leakage points within or around areas of serous retinal detachment. In acute cases, leakage mostly originates from a single site and can create patterns like the “inkblot” pattern, “smokestack” pattern, and diffuse circular hyperfluorescence pattern in later stages [16]. In addition, point leaks can often be detected in the macula of diseased eyes [17]. In contrast, leakage in chronic CSCR is mostly from multiple sites and shows granular hyperfluorescence on FA [18,19].

ICGA imaging of choroidal vasculature was introduced in the early 1990s [6]. Although FA is more effective than ICGA in showing leakage sites in the acute phase of the disease, it has been noted that the hyperfluorescence on ICGA persists long after leakage ceases on FA. Typical findings on ICGA include mild hyperfluorescence during the early phase, variable hyperfluorescence during mid-phase, and marked hyperfluorescence in late phase of CSCR [6,20]. In both FA and ICGA, about 80% of dye binds to plasma, and the unbound dye in the chorioretinal vessels provides spatial contrast between the vasculature and its surroundings. However, retina pathologies require that we understand the metabolic effect of red blood cell flow in the choroid vasculature because of established choroidal hyperpermeability in diseased eyes. Retina vasomotion was described by Flower, et al. using ICG-loaded erythrocyte ghost cells to address this issue and enhance understanding of retina edema in pathologies like CSCR [21].

On fundus autofluorescence (FAF), a decreased or absent autofluorescent signal can be seen, which indicates reduced metabolic activity of the RPE, mainly due to photoreceptor loss. Dark hypoautofluorescent descending tracts can also be seen, which reflect atrophic patterns of chronic fluid accumulation [22]. Autofluorescence could be useful in understanding the extent of the disease and prognosis.

AO-SLO has been used in the detection and diagnosis of CSCR. This technique has evolved to provide detailed cellular imaging of the retina [23]. AO-SLO has the advantage of using a confocal configuration to generate images at different planes and depths in the retina [24,25,26]. This feature enables the visualization of photoreceptor mosaics, retinal nerve fiber layer, Henle fiber layer, blood vessels and capillary imaging in CSCR [27,28,29,30]. AO-SLO is an imaging tool that can be used to evaluate retinal layer integrity and changes in visual outcomes before and after resolution of CSCR. It can be combined with other devices such as fundus camera and OCT to detect changes in photoreceptor mosaic pattern in patients with CSCR [31,32]. The AO-SLO is able to detect decrease in cone density and disruption of inner segment/outer segment (IS/OS) or intermediate line on OCT [33]. The photoreceptor alteration can be attributed to failure of metabolic support to photoreceptors due to RPE damage and excess fluid from choroidal hyperpermeability. AO-SLO can provide precise localization of intraretinal structures such as hyperreflective dots (HRDs) which represent cellular debris from serous detachment of RPE and decreased retinal metabolic support [34].

SD-OCT has emerged as the primary imaging modality in the diagnosis of CSCR [6]. It is a reproducible, non-invasive imaging modality that provides high definition anatomical and functional images of the retina and choroid to identify pathologies such as CSCR [35,36]. In CSCR, subretinal fluid accumulation due to disruption of the RPE barrier and choroidal hyperpermeability can be detected on OCT. This is seen on OCT as separation of the neurosensory retina and RPE at the macula [37]. This technique has been used to detect increased choroidal thickness in patients with CSCR [38].

There are limited studies on the sensitivity and specificity of the various choroidal imaging techniques to differentiate CSCR from other chorioretinal pathologies that cause macular edema. Future studies are needed to clarify the constellation of features or biomarkers on each imaging technique that can definitively diagnose CSCR.

This review article aims to provide an update on choroidal imaging and how imaging can help determine the pathogenesis and treatment for CSCR.

## 2. Methods

A systematic literature search was conducted using Ovid, EBSCO, MEDLINE and PubMed (1977 to 30 June 2020). The search terms included (1) “central serous chorioretinopathy”, (2) “central serous chorioretinopathy biomarkers”, (3) “central serous chorioretinopathy imaging, (4) “central serous chorioretinopathy autofluorescence”, (5) “choriocapillaris”, (6) “OCT angiography (OCTA) and the choroid”. Only articles reporting on original clinical data were selected, studies in a language other than English were included only if an English abstract was provided. Additional sources included articles cited in the references list of the first selected articles. The following information was extracted from the selected studies: description of disease, similarities and differences when compared to central serous chorioretinopathy, clinical features, and choroidal findings from AO-SLO, FA, ICGA, FAF, OCT and OCTA.

## 3. Results/Findings

### 3.1. Choroidal Layer Thickness in CSCR

Increased choroidal thickness in eyes with CSCR has been reported in the literature [39,40] and is thought to be due to dilation of large choroidal vessels [9]. It has also been found that dilated vessels are mostly in areas of high choroidal vascular permeability on ICGA. However, in a study of 173 patients by Rijssen, et al., there were no statistically significant differences in choroidal thickness between affected eyes and fellow eyes in untreated chronic CSCR [41]. This supports the bilateralism of CSCR in the literature [42,43]. Therefore, choroidal thickness is increased in both the affected and fellow eye. Although choroidal thickness is associated with CSCR, it is not necessary for its diagnosis as different patient factors like age, myopia and axial length can result in decreased choroidal thickness [6].

There is increased choroidal thickness among eyes with CSCR compared to healthy eyes. In a recent cohort study to evaluate choroidal layer thickness in CSCR, subfoveal choroidal thickness (SFCT) as well as medium and large choroidal vessel thickness were increased in eyes with acute CSCR compared to healthy control eyes (acute 360 ± 94.7 µm, chronic 338.4 ± 86.9 µm, normal eye 277.7 ± 46.8 µm; *p* = 0.0001) [44]. The mean choroidal vessel thickness elevation was significant at 750 µm nasal to the fovea in acute (*p* = 0.006) or chronic (*p* = 0.008) CSCR eyes compared to the control group while an insignificant difference was found in measurements 750 μm temporal to the fovea. These findings suggest that changes in choroidal thickness findings in CSCR may not be uniform; therefore, it is imperative to assess choroidal thickness from the same locus during follow-up visits.

The increase in choroidal thickness has been attributed to subretinal fluid leakage and choroidal interstitial edema. One study found a significant reduction in large choroidal vessel diameter under the leak after resolution of subretinal fluid. It was noted that resolution of subretinal fluid contributed to less than a 50% reduction in Haller’s layer thickness. Therefore, choroidal interstitial edema likely plays a major role in increasing choroidal thickness seen in CSCR [45].

OCT findings have shown increased choroidal thickness in affected and fellow eyes which again reiterates that the fellow eyes of the CSCR patients should not be considered as “normal”.

### 3.2. Choroidal Vascularity Index (CVI) in CSCR

**Figure 2:**
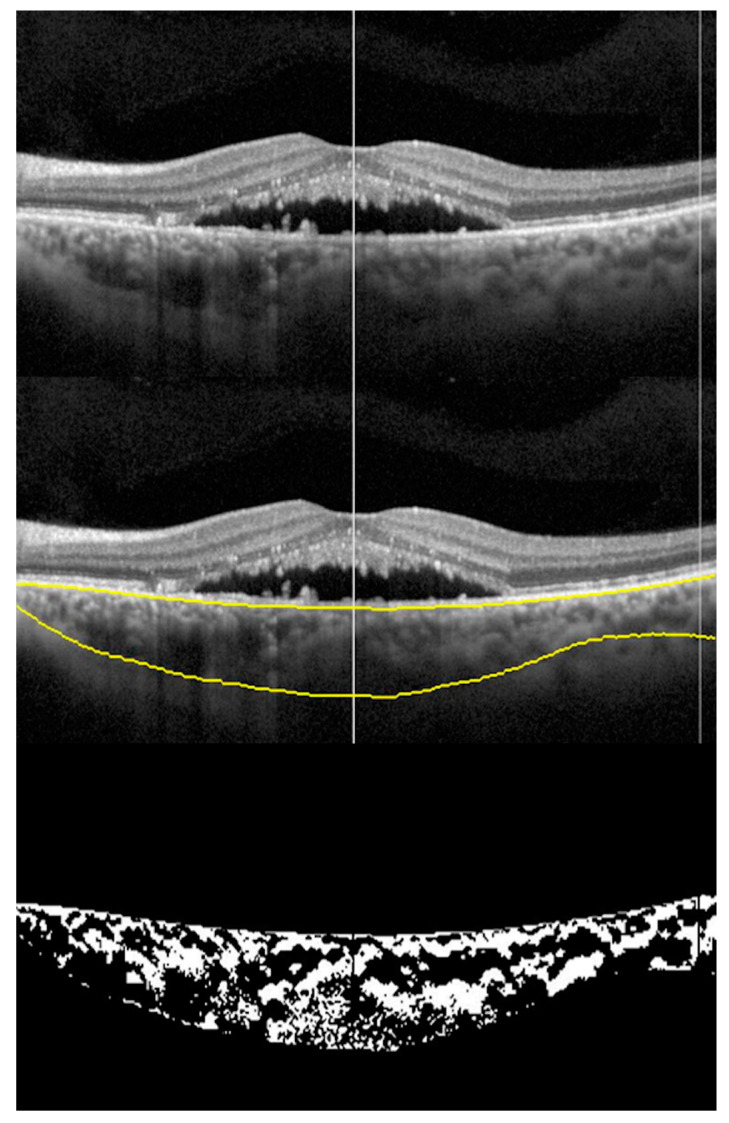
Choroidal vascularity Index (CVI): montage showing the process of CVI calculation. Top scan shows neurosensory detachment in an eye with CSCR; middle scan shows choroidal boundaries using automated algorithm; and bottom scan shows the binarized image of the choroid with CVI of 0.6491.

Abnormalities in the choroid, which comprises mostly blood vessels and interstitial stroma is involved in the pathogenesis of CSCR. CVI has emerged as an imaging biomarker for CSCR. In a recent study of 78 eyes (39 patients) with acute or resolved CSCR, CVI` was calculated from enhanced depth imaging OCT scans (Figure 2). There was a higher CVI and SFCT in eyes with active disease compared to eyes with resolved disease, normal fellow eyes, and age-matched healthy eyes (CVI: 70.54 ± 0.15, 65.14 ± 0.1, 67.42 ± 0.08, 65.18 ± 0.2, *p* < 0.0001; SFCT: 474.63 ± 148.52, 374.6 ± 65.13, 401.63 ± 124.30, 278.5 ± 65.31; in active, resolved, normal fellow eyes, and normal control eyes respectively, *p* < 0.0001). There was a significant increase in CVI in the acute diseased state compared to fellow eyes (*p* < 0.0001). However, CVI in fellow eyes was also increased compared to age-matched healthy eyes (*p* < 0.0001). The increase in CVI of fellow eyes reiterates the bilateralism of CSCR [13]. Enhanced depth imaging OCT scans provide the depth of choroidal features, which is useful for calculating CVI in patients with CSCR. A change in CVI may suggest a resolution of disease or positive treatment response.

#### Choroidal Vascularity Changes in Acute CSCR

Acute CSCR can be a self-limiting disease [46], hence observation is usually the first approach [47]. Here, we look at the choroidal vascularity changes during the acute phase with or without treatment. One study compared the effect of laser photocoagulation on choroidal vascularity changes using spectral domain optical coherence tomography scans in both the laser and control treatment groups. All parameters (central macula thickness, neurosensory detachment, subfoveal choroidal thickness, and best corrected visual acuity) showed statistically significant improvement in both treatment and control groups at multiple follow-up visits (one-month, three-month, six-month) [48].

Laser photocoagulation has not been shown to significantly affect the increased CVI in the acute phase. In a recent study of 30 patients, there was a statistically significant increase in CVI in the control group compared to the laser group at three months and six months. However, at 6 months, there was no significant increase in CVI between groups. Early laser treatment showed no significant morphological changes in the choroid compared to observation; therefore, there is no current data to suggest added benefit in the acute phase [49]. This study supports the initial observation that there is no benefit on choroidal morphology after early laser photocoagulation in acute CSCR. For patients with non-resolving, recurrent and chronic CSCR, available treatments include; half dose photodynamic therapy, subthreshold micropulse laser, MR antagonist and transpupillary thermal therapy [50].

### 3.3. Wide Field Choroidal Vascularity and Choroidal Thickness in Healthy and CSCR

**Figure 3:**
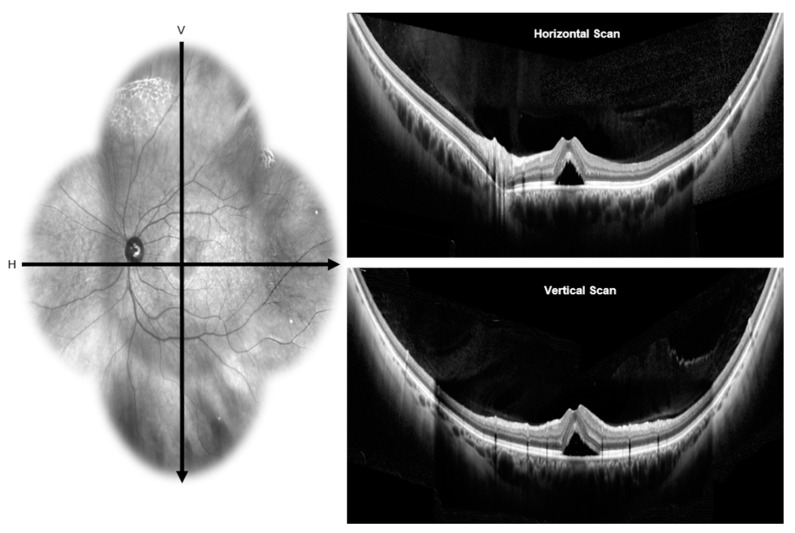
Wide-field optical coherence tomography: the infra-red image on the left side showing the horizontal and vertical scans passing through the fovea of an eye with CSCR. Wide-field optical coherence tomography scans on the right side shows presence of neurosensory detachment in horizontal (top) and vertical (bottom) scans.

Historically, OCT has provided quasi-histologic sections of the retina in vivo [51,52]. Advances in OCT has led to enhanced depth imaging (EDI) [53] and swept-source types [54] which provided a clearer visualization of the retina, choroid, and sclera. EDI sets the choroid adjacent to the zero-delay line, which is the axial range position of maximal sensitivity for signal detection, to produce detailed choroidal images through an undilated pupil [55]. EDI produces an inverted image with well-focused illumination at the level of the choroid or the inner sclera [55]. Swept source technology employs a light source of about 1 µm that sweeps across a narrow band of wavelengths (longer wavelength light source e.g., at 1050 nm) to improve visualization of deeper structures beneath the RPE (particularly the choroid) by decreasing sensitivity roll off and attenuation of signals [56]. With the introduction of wide-field OCT imaging modalities, the structural details of the perimacular and peripheral retina along with the choroid are accessible. Wide field imaging provides a field of view of 80 degrees in primary gaze up to 200 degrees in both the vertical and horizontal meridians using manual montage [57,58,59,60] (Figure 3).

The CVI of healthy eyes taken by wide-field choroidal images using swept-source OCT showed the macula as the area with the least CVI (40.01 ± 7.67, *p* < 0.01). The highest was found in the nasal region (50.84 ± 5.64) followed by the inferior > temporal > superior quadrants [61]. CVI values for nasal, macula, and temporal segments were calculated from horizontal scans while superior, and inferior segments were calculated from vertical scans. The unit of measurement was defined as single unit equals the distance between the center of the optic disc and the fovea. Macula area in horizontal and vertical meridian scans were defined as two disc-to fovea distance units (one on either side of fovea) in horizontal and vertical scans respectively. The superior region had the highest variability, and the nasal segment had the lowest variability. Of the factors that could affect CVI (age, sex, refraction, intraocular pressure, axial length, mean arterial pressure and mean ocular perfusion pressure), only age had a significant negative effect on CVI (coefficient −0.004). A possible explanation for the low CVI and high choroidal thickness in the macula area is due to increased interstitial stroma and increased area. The differential ratio of large to small vessels in the macula area compared to the peripheral regions account for the low CVI. Also, the macula has a much higher number of medium vessels and thicker choriocapillaris, which contribute to the low CVI. A high number of thick vessels and less stroma contribute to high CVI [61].

A regional variation in choroidal thickness and large choroidal vessel thickness up to the mid-equator using the swept- source OCT has also been described. The mean choroidal thickness in the vertical scans passing through the fovea was higher than the horizontal scans (331.2 ± 76.34, vs. 245.79 ± 55.38). Choroidal thickness was significantly reduced at all peripheral points (temporal > nasal > superior > inferior) compared to SFCT (*p* < 0.05). The mean large choroidal vessel thickness was significantly higher in the vertical segment compared to the horizontal macular segment (201.46 ± 54.31 vs. 150.38 ± 52.58). The reduced choroidal thickness and large choroidal vessel thickness in horizontal macular segment compared to the vertical segment were due to the inclusion of peripapillary area in horizontal segments where the choroid thinning occurs before termination at the optic disc. In both the vertical and horizontal meridians, the macular region had the highest choroidal thickness and large choroidal vessel thickness. It is assumed that the high metabolic demand of the macula is met exclusively by the choroid [58].

In patients with CSCR, the mean SFCT was higher at the macular level (478 ± 114 µm), 367 ± 94 µm in the superior periphery, 257 ± 103 µm in the inferior periphery, 431 ± 121 in the nasal and 280 ± 88 µm in the temporal periphery [60]. Wide-field images showed relative thinning of the inner choroidal layer in the periphery. The small and medium-large vessels ranged from 86 µm nasally to 120.1 µm superiorly, with a mean of 98.8 ± 13.6 µm. The outer choroidal layer thickness ranged from 175.5 µm temporally to 235.5 µm superiorly, with a mean of 217.8 ± 41.4 µm [60].

CVI and choroidal thickness are useful early indicators for the diagnosis of CSCR and monitoring of treatment response. Wide field imaging provides a great peripheral view for comparison and understanding of peripheral choroid.

### 3.4. En Face Enhanced-Depth Swept Source-Optical Coherence Tomography Features in CSCR

En face OCT imaging is an emerging technology that combines spectral domain technique with transverse confocal analysis to produce transverse images of retinal and choroidal layers at specified depth [62]. Special purpose software has also been used to generate en face projection images based on reflectance intensity for a fixed slab of OCT cube scans [63]. The en face technique requires high-speed acquisition scans because each pixel requires an axial scan with high quality vascular anatomy images of individual choroidal layers and choriocapillaris [64].

The evaluation of the RPE and choroid in eyes with CSCR using en face enhanced-depth swept source-OCT has demonstrated absence of signal at the RPE corresponding to RPE detachment and/or loss in all eyes. In a study of fifteen (15) eyes with CSCR, more than half (53% of all eyes) had focally enlarged vessels at the choriocapillaris layer and Sattler’s layer, while 47% showed diffuse vessel dilation at the Sattler’s layer 64. At the level of Haller’s layer, 20% were focal dilations with 80% of the vessels diffusely dilated. In the above study, en face images of choroid and RPE were extracted at varying depths every 3.5 µm and analyzed for abnormal features using multimodal imaging like FAF, FA and ICGA. A pattern of local or diffuse choroidal dilation was made manually based on similarities between multiple imaging modalities [64].

#### En Face Choroidal Vascularity in CSCR

**Figure 4:**
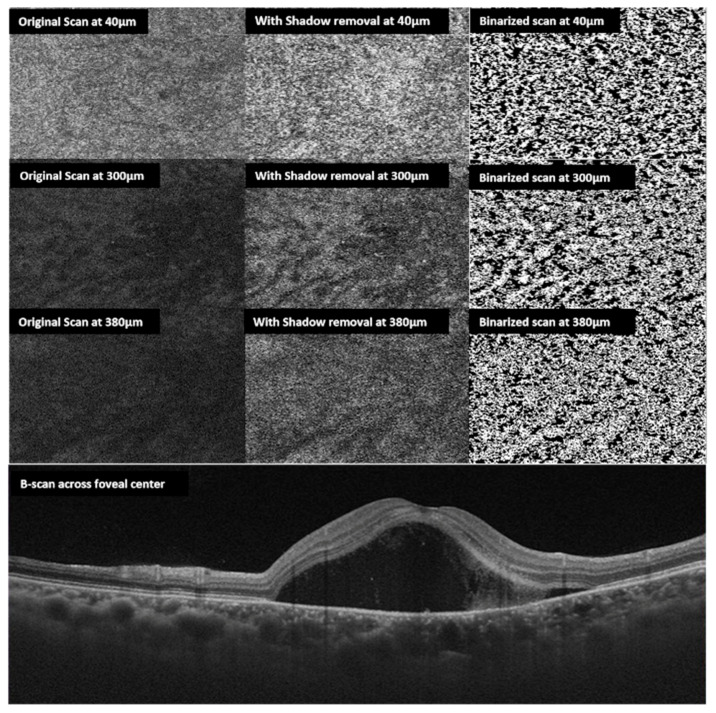
En face choroidal vascularity: multiple en face scans obtained at 40, 300, 380 µm from the Bruch’s membrane top to bottom respectively (left side). The middle column shows en-face scans following shadow removal. The right column shows corresponding binarized scans. The bottom horizontal B scan shows the neurosensory detachment of the same eye with CSCR.

**Figure 5:**
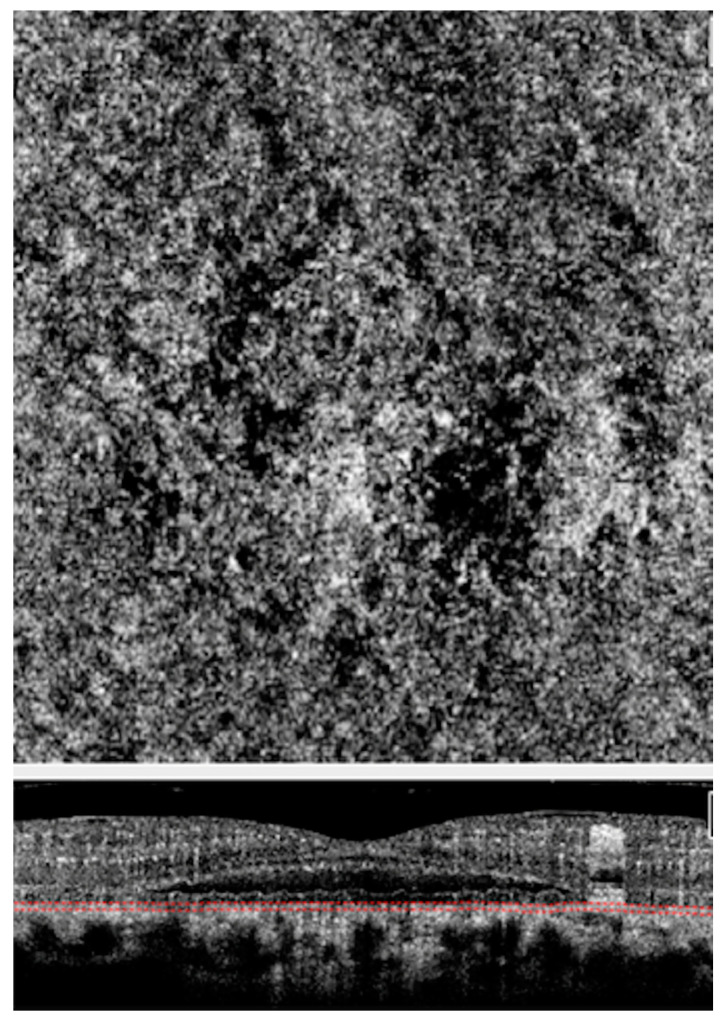
Optical coherence tomography angiography (OCTA) of an eye with CSCR. Choriocapillaris slab (**Above**) of OCTA scan above showing choriocapillaris flow void areas (dark areas) and corresponding cross-sectional scan (**Below**) showing the scan location at the choriocapillaris level.

Algorithms have been developed to measure choroidal vascularity of en face OCT scan by extraction and binarization. Vupparaboina, et al. demonstrated an automated vascularity estimation technique. The technique uses scans separated by 5 µm segments of choroid based on a validated algorithm in which RPE-Bruch’s complex and choroid scleral interface were identified using structural similarity index, Hessian analysis and tensor voting [65]. An adaptive histogram equalization method is then employed to enhance contrast between choroidal stroma and blood vessel lumen. In addition, binarized images are reviewed by independent observers blinded to each other to make sure the images are correctly converted by comparison with the original en face scans [66] (Figure 4 and Figure 5).

The CVI of CSCR patients increased steadily beyond the Bruch’s membrane; maximum at 75% of choroid in acute disease and 50% of choroid in chronic disease [65]. The mean CVI of several en-face sections 5 μm apart were not statistically significantly different between acute and chronic CSCR (47.97 ± 1.25 for acute and 48.24 ± 1.94 for chronic.) Using a post hoc test with Bonferroni correction, the mean CVI at 25%, 33%, 50%, 75% depths of choroid were significantly larger than the mean CVI at the choriocapillaris and choroidoscleral interface (*p* < 0.001) for both acute and chronic patients. This shows similar trends in CVI increasing towards maximum at the mid choroid and similar values at different depths of the choroid in both acute and chronic CSCR. Choroidal status looks similar in both acute and chronic disease [66].

As described earlier, there is dilatation of Haller’s layer, which is the layer of outer large choroidal vessels, and attenuation of Sattler’s layer and the choriocapillaris in a diseased state. In a retrospective study of 30 eyes, Wong et al. showed the mean CVI of eyes with acute disease was 45.21% ± 2.25% at the choriocapillaris, which increased to the maximal value of 48.35% ± 2.06% at 75% depth of the choroidal thickness and 45.31% ± 3.27% at the choroidoscleral interface [66]. In eyes with chronic disease, the mean CVI was 44.76% ± 2.60% at the choriocapillaris, which hit a maximum at 50% choroidal depth (48.70% ± 1.32%) and then returned to 45.41% ± 6.02% at the choroidoscleral interface. There was dilatation of the Haller’s layer in CSCSR, which is hypothesized to cause compression of the inner choroidal smaller vessels resulting in leakage and accumulation of serous fluid under the retina [66].

Morphological differences in Haller’s vessel arrangement in healthy, acute and chronic eyes have been studied using en face optical coherence tomography. The prevalence of “herringbone pattern” in healthy eyes (49.2%) and “reticular pattern” in both acute and chronic (combined 48.8%) has been described [60,67,68]. These patterns were coined by Savastano, et al. based on images from en face choroidal scans [68]. There is a similar pattern distribution for both acute and chronic CSCR, suggesting en face OCT has a formal role in classification of choroidal disease such as CSCR. Structural en-face OCT helps visualize patchy vessels and confirms the choroidal changes using non-invasive imaging.

### 3.5. Choroidal Vascular Reactivity in CSCR

Hypertensive patients have a high risk of CSCR (Odds ratio: 2.25–2.3) [69]. Compared to age-matched healthy cohorts, patients with CSCR were also found to have a sympathetic-parasympathetic imbalance [6]. In a study to assess the choroidal vascular response to experimentally increased blood pressure in patients with CSCR using OCTA, there was increased vascular density (*p* < 0.005) in patients with CSCR under stress conditions although they had a lower baseline vascular density values than controls. The increased vascular density was hypothesized to be due to dilation of vessels not angiogenesis [70].

The physiological mechanisms regulating homeostasis in choroidal circulation in response to stress may be dysfunctional in patients with CSCR. Also, a neural or vascular endothelial autoregulation dysfunction [71] and myogenic regulatory mechanism failure [72] can explain the instantaneous response to blood pressure changes. Choroidal thickness may be partially controlled by nonvascular smooth muscle cells, which are under both sympathetic and parasympathetic innervation. Since thinning of the choroid occurs under parasympathetic stimuli, sympathetic overactivation and nonvascular smooth muscle cell relaxation may play a role in choroidal thickening in CSCR [73,74].

### 3.6. Choriocapillaris on OCT and OCTA in CSCR

There is increased hyperpermeability in the lobules of the choriocapillaris resulting from congestion of the choroidal vortex veins in patients with CSCR [75]. In a retrospective study to characterize outer choroidal vessels in normal and CSCR eyes, the authors found dominant veins were dilated markedly in disease state. All diseased eyes had asymmetric dominant vortex veins compared to 38% in normal eyes. En face choroidal images in diseased eyes showed no distinct vascular pattern at the level of the choriocapillaris. However, there was moderate dilation at Sattler’s layer and marked dilation at Haller’s layer. The authors hypothesized that hyperpermeability occurs in lobules of the choriocapillaris when these collecting venules drain into the branches of the congested vortex veins [75]. The congested dominant vein retard arterial blood flow causing leakage.

OCTA is the only method enabling noninvasive visualization of choriocapillaris [76]. This angiographic technique is able to detect vessels with moving blood cells, and blood cell flow changes at the level of the choriocapillaris. A specific finding associated with alteration of choriocapillaris is “flow voids,” which reflects lack of detectable blood cell velocity in the choriocapillaris. There is increased total flow void area in disease eyes compared to age-matched controls [76]. Additionally, increase of total flow voids area increases from acute to recurrent and, further, to chronic, in other words with an increase of disease severity [76].

These flow voids increase in number with age and demonstrate a specific spatial distribution, above the large choroidal vessels. The flow voids are common in CSCR but not in healthy eyes [77]. The flow voids are created by focal compression of the choriocapillaris by large choroidal vessels resulting in choriocapillaris perfusion deficit followed by retinal pigment epithelium alteration [77]. Similar studies have shown higher abundance of flow void in eyes with pachychoroid pigment epitheliopathy in addition to decreased choriocapillaris vascular density compared with controls [78]. Further analysis of the location of these flow voids, their effect at the cellular level and contribution to fluid accumulation in the retina are needed to better understand the pathogenesis of CSCR.

Several reports exist regarding the OCTA characteristics of CSCR. OCTA is extremely accurate in detecting neovascular membranes that appear in chronic CSCR and has shown to be superior over conventional imaging modalities [79]. A rare fraction area in the choriocapillaris can be observed that may correspond to flow void due to focal atrophy of the choriocapillaris secondary to compression by the enlarged vessels of Haller’s layer or that reflects a lack of detectable blood cell velocity [80]. Recently, localized areas of increased and decreased OCTA signal in acute CSCR represents evidence of choriocapillaris inflammation with enhanced flow in this area, and surrounding decreased flow in areas of choriocapillaris atrophy. After resolution of acute CSCR, OCTA findings return to normal, which suggests choriocapillaris architecture normalization [81].

### 3.7. Retinal and Choroidal Hyper-Reflective Dots (HRDs) in CSCR 

**Figure 6:**
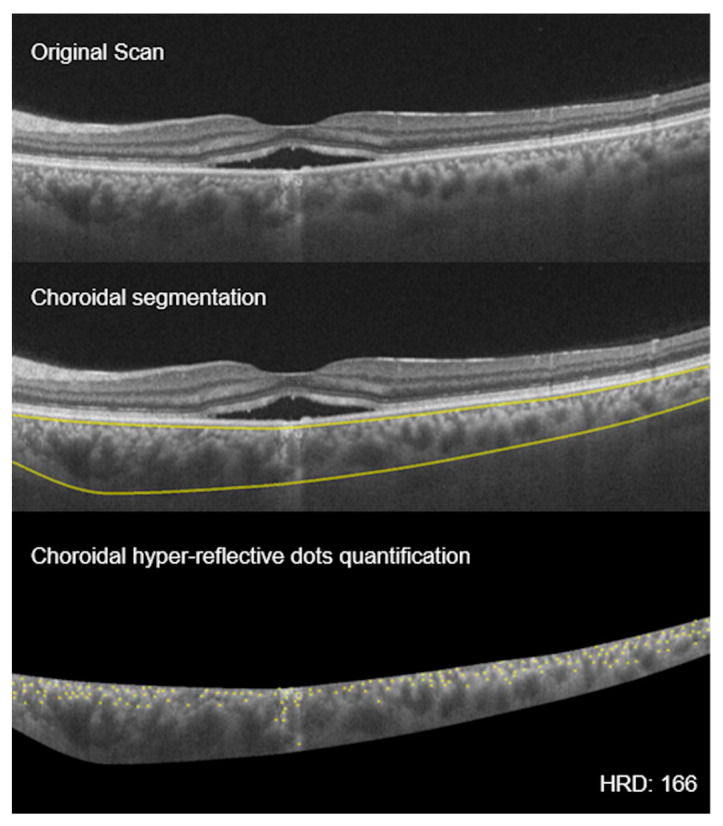
Choroidal hyperreflective dots: montage showing the process of choroidal hyperreflective dots (HRD) calculation. **Top** scan shows neurosensory detachment in an eye with CSCR; **middle** scan shows choroidal boundaries using automated algorithm; and **bottom** scan shows the yellow hyperreflective dots of the choroid (HRD = 166).

HRDs are correlated with SFCT, central macula thickness, and height of neurosensory detachment [82]. The mean count of choroidal HRDs in acute disease was significantly higher than chronic disease (acute = 139.4 ± 29.9 and chronic = 124.9 ± 28.1 respectively (*p* = 0.04)). HRDs were negatively correlated with age and SFCT in all subjects. Mean duration of disease had no correlation with number HRDs. Although the exact cellular origin of HRDs are unknown, its presence in CSCR has been attributed to inflammatory response in the retina and choroid. The higher HRD counts in acute disease might be as a result of ongoing neurosensory detachment [82] (Figure 6). Although AO-SLO imaging discussed earlier shows localization of intraretinal HRDs, quantification methods have not been established. Larger studies with longitudinal follow-up using AOSLO quantification of HRDs are needed to determine their clinical significance and relationship to the pathogenesis and resolution of CSCR.

### 3.8. Haller Vessel Segmentation

**Figure 7:**
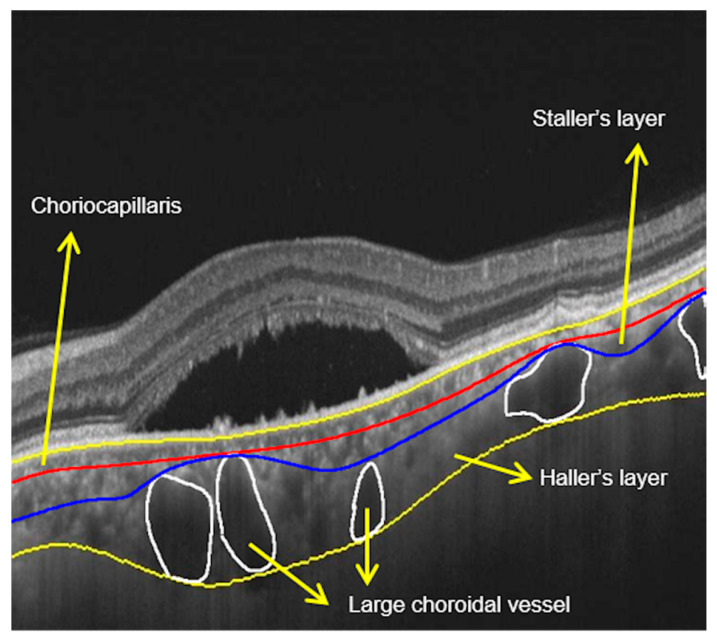
Haller’s layer measurements: scan showing neurosensory detachment with demarcation of various layers of choroid. Large choroidal vessels are marked, and three layers of the choroid are marked.

Automated quantification of choriocapillaris, Haller’s layer, and Sattler’s layer thickness is an important biomarker for the pathogenesis of CSCR. In a study comparing the thickness of the choriocapillaris, Sattler’s layer, and Haller’s layer with EDI-OCT, the authors found statistically significant thicker Haller’s layer in diseased eyes versus fellow eye (disease patients) and eyes of healthy individuals; there were no differences in choriocapillaris/Sattler layer between study groups [83]. This finding suggests that Haller’s layer is the main contributor to choroidal thickening in CSCR. Haller’s layer thickness may be a more sensitive biomarker for disease activity than total choroidal thickness. However, the measurements were performed manually and were associated with relatively low interobserver reliability of 0.795 [12]. An automated quantification of Haller’s layer is important for further investigations on choroidal layer changes in CSCR (Figure 7). A study using an algorithm validated both quantitatively and qualitatively was able to delineate the different layers of the choroid [83]. Unlike retinal layers, choroidal layers are not separated by sharp intensity transitions, making delineation a challenge. Because of this challenge, traditional gradient based techniques cannot be employed. A study employed algorithm using extended-minimal transform alongside watershed segmentation to address this challenge. The algorithm was able to achieve delineation results with a dice coefficient of 89.48% and a high intra-observer dice coefficient of 89.12%. The absolute difference for automated vs. manual evaluation was 17.54% and 19.9%, respectively. The higher correlation between the algorithmic and manual delineation indicates the possibility of clinically analyzing choroid in finer detail, especially in disease state [83]. Haller vessel analysis explores the pachychroid mechanism in CSCR and may help understand progression of the disease process with inner choroidal thinning and chronic complications.

## 4. Future Research Directions and Limitations

Choroidal imaging has advanced, and upcoming in-vivo choroidal imaging techniques have improved visualization almost up to the microscopic level. Application of adaptive optics-indocyanine green angiography (AO-ICG) has been recently been employed to visualize choriocapillaris and outer retina at the cellular level [84]. Laser doppler holography is the most recent advanced non-invasive imaging used to visualize the choroidal vasculature. Holography analysis calculates the best spectrum between a doppler broadened beam and a monochromatic reference beam [85]. This device provides better images of the choroidal vessels compared to conventional ICG. In addition, it differentiates choroidal arteries and veins, which is not possible on ICG [86]. Such updated imaging techniques to visualize choriocapillaris and large choroidal vessels will further improve our understanding of pachychoroid diseases, particularly CSCR.

This review article was limited by our inability to include all relevant current choroidal findings in CSCR from the search database (Ovid, EBSCO, MEDLINE and PubMed (1977 to 30 June 2020).

## 5. Conclusions

Imaging modalities such as FA, ICGA, spectral domain and swept source OCT, and OCTA have advanced the diagnosis of CSCR. This has led to numerous imaging biomarkers for early detection and prognostication of CSCR. Key amongst these imaging markers are CVI, choroidal thickness, and HRDs. Currently, imaging techniques like the wide field OCT and en face OCT used in the diagnosis of CSCR can be employed to thoroughly evaluate asymptomatic fellow eyes to guide treatment options. This review paper on the current state of imaging in CSCR gives a framework of the characteristic/relevant imaging findings of eyes with both acute and chronic CSCR in addition to fellow eyes.
